# Paintings by Turner and Monet depict trends in 19th century air pollution

**DOI:** 10.1073/pnas.2219118120

**Published:** 2023-01-31

**Authors:** Anna Lea Albright, Peter Huybers

**Affiliations:** ^a^Laboratory of Dynamic Meteorology, Sorbonne University, École normale supérieure, Paris 75005, France; ^b^Department of Earth and Planetary Sciences, Harvard University, Cambridge, MA 02138

**Keywords:** air pollution, artwork, environmental reconstruction, atmospheric science

## Abstract

Individual paintings by artists including Vincent van Gogh and Edvard Munch have been shown to depict specific atmospheric phenomena, raising the question of whether longer-term environmental change influences stylistic trends in painting. Anthropogenic aerosol emissions increased to unprecedented levels during the 19th century as a consequence of the Industrial Revolution, particularly in Western European cities, leading to an optical environment having less contrast and more intensity. Here, we show that trends from more figurative to impressionistic representations in J.M.W. Turner and Claude Monet’s paintings in London and Paris over the 19th century accurately render physical changes in their local optical environment. In particular, we demonstrate that changes in local sulfur dioxide emissions are a highly statistically significant explanatory variable for trends in the contrast and intensity of Turner, Monet, and others’ works, including after controlling for time trends and subject matter. Industrialization altered the environmental context in which Turner and Monet painted, and our results indicate that their paintings capture changes in the optical environment associated with increasingly polluted atmospheres during the Industrial Revolution.

## Significance

Individual paintings are known to depict snapshots of particular atmospheric phenomena, raising the possibility that paintings could also document longer-term environmental change. During the Industrial Revolution, air pollution increased to unprecedented levels, but these values remain uncertain given the lack of widespread, direct measurements. Here, we show that stylistic changes from more figurative to impressionistic paintings by Turner and Monet over the 19th century strongly covary with increasing levels of air pollution. In particular, stylistic changes in their work toward hazier contours and a whiter color palette are consistent with the optical changes expected from higher atmospheric aerosol concentrations. These results indicate that Turner and Monet’s paintings capture elements of the atmospheric environmental transformation during the Industrial Revolution.

Some works of art, even those that do not appear “realistic,” appear to faithfully record particular natural phenomena. Edvard Munch’s *The Scream* (1893), for example, is argued to depict nacreous clouds (1). Vincent van Gogh’s *Moonrise* (1889) is dated to precisely 9:08 p.m. local time on July 13, 1889, using topographic observations, lunar tables, and letters (2). Nine of Claude Monet’s paintings in his London series are also dated using solar geometry, with results confirmed by cross-referencing against Monet’s letters (3). A survey of over 12,000 paintings, moreover, indicates that different schools reflect local meteorological conditions, such as paler blue skies in the British school than other contemporaneous European schools (4). Another important example of paintings depicting the natural environment comes from a set of studies of sunset coloration over time relative to volcanic eruptions that injected aerosols into the stratosphere (5, 6). Sunsets seen through an aerosol-laden stratosphere appear redder because of greater scattering in the limb of the Earth’s atmosphere (7). Across schools of painting, the red-to-green ratios in sunset paintings from 1500 to 1900 are correlated with independent proxies of stratospheric aerosol content (5, 6), though difficulty constraining the aerosol size distribution and solar zenith angle introduces uncertainties to this methodology (8).

Here, we seek to ascertain whether there is a relationship between changes in atmospheric conditions associated with industrialization and changes in painting style—primarily that of the British artist Joseph Mallord William Turner (1775 to 1851) and French artist Claude Monet (1840 to 1926). We focus on Turner and Monet because they prolifically painted landscapes and cityscapes, often with repeated motifs. Furthermore, Turner and Monet’s works span the Industrial Revolutions starting in Great Britain in the late 18th century, a time of unprecedented growth in air pollution (9–11). Over the course of their careers, Turner and Monet’s painting styles change from sharper to hazier contours and toward a whiter palette, a progression that is typically characterized as moving from a more figurative to impressionistic style. We explore the hypothesis that increasingly impressionistic paintings by Turner, Monet, and several other artists represent, at least in part, physical changes in atmospheric optical conditions.

## Optical Implications of Increasing Aerosol Concentrations

**Fig. 1.**
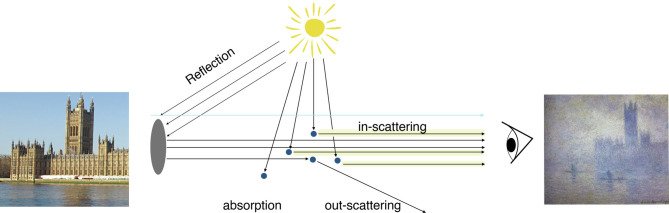
Schematic illustrating key processes by which aerosols influence an object’s contrast, intensity, and visibility. A theoretical object (denoted by the gray disk) reflecting light (black arrows) is visible because of its contrast with the background light (pale blue arrow). Aerosols (navy dots) in the air column scatter background light into the line of vision (“in-scattering,” highlighted in light yellow), scatter object light out of the line of vision (“out-scattering”), and absorb light. These optical effects from aerosols lead a viewer to perceive an object as having less-distinct edges (less contrast) and a whiter tint (increased intensity), as idealized by the images on the left- and right-hand side (Claude Monet’s *Houses of Parliament, Effect of Fog*, 1899–1904) and described in *Methods*.

As illustrated in Fig. 1, aerosols absorb and scatter radiation both into and out of a line of sight. This scattering tends to decrease the contrast between otherwise distinct objects (12, 13). Edges are used to quantify contrast because they often show the intensity

of an object in the foreground relative to that of the background along nearly equal lines of sight. In order to objectively define contrast in a manner that adapts to the scale and perspective of an image, we use a wavelet technique. Wavelet analysis is selected over Fourier analysis because it allows for quantifying the local contrast in images (14), and was previously used to estimate visibility in urban photographic images (15). We use a Haar wavelet whereby first differences of an image are taken at various scales (16), ranging from individual pixels to spanning the height or width of an image. An index of the contrast found in an image is obtained by computing the 95th percentile of the wavelet coefficients, *w*_95_, normalizing by the median value, *w*_50_, and taking the logarithm,

[1] $$\begin{array}{c} \;\text{contrast index}=\log(w_{95}/w_{50}). \end{array}$$

Normalization accounts for different baseline edge strengths depending on lighting, scene, and image resolution, and the logarithm is suggested by the exponential dependence of contrast on the extinction coefficient (*Methods*). *SI Appendix*, Fig. S1 shows four example paintings illustrating that the largest gradients relate to distinct features, such as waves, a bridge, and the hull of ships.

### Benchmarking with Photographs.

We first demonstrate our metric for contrast on pairs of photographs taken during clear and polluted conditions (*SI Appendix*, Fig. S2). These photographic pairs involve less artistic interpretation and allow for benchmarking our technique using better-controlled image characteristics. Consistent with our expectations, every polluted photograph has a lower contrast index than its clear-sky counterpart (*SI Appendix*, Fig. S2). The mean fractional reduction in the contrast index from clear-sky to polluted photographs is 19%. The same techniques used for photographs are next applied to evaluate trends in contrast in paintings, which are then evaluated in relation to aerosol emissions over time.

### Quantifying Historical Air Pollution.

**Fig. 2.**
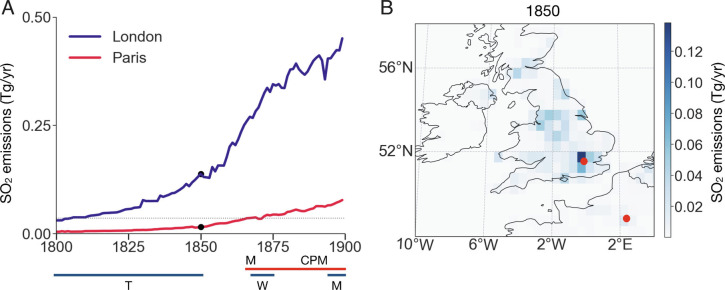
19th century sulfur dioxide (SO_2_) emissions in London and Paris. (*A*) Time series of emissions (17) in the grid boxes encompassing London (blue) and Paris (red). Years of paintings by Turner (T), Monet (M), Whistler (W), and Caillebotte, Pissarro, and Morisot (CPM) are indicated by horizontal lines for London (blue) and Paris (red). Emissions during Monet’s early paintings correspond to those of Turner’s early paintings. (The dotted black line represents mean Parisian emissions from 1864 to 1872). (*B*) A geographic distribution of SO_2_ emissions in 1850, highlighting how emissions are concentrated in London (red point in England) and that emissions in Paris (red point in France around 2°E) trail those in London.

As a proxy for historic variations in anthropogenic aerosol concentrations, we use a gridded estimate of annual emissions of sulfur dioxide, SO_2_ (17). The early Industrial Revolution was largely powered by coal (11, 18), and coal typically contains 1 to 5% sulfur by dry weight (19). From 1800 to 1850, the United Kingdom emitted nearly half of global SO_2_ emissions, and the grid box corresponding to London, known as the “Big Smoke” (9–11), accounts for approximately 10% of all UK SO_2_ emissions (Fig. 2), despite accounting for only 1.0% of the area. *SI Appendix*, Fig. S3 presents qualitative evidence for the optical effects associated with historical London air pollution captured by sketches and photographs.

SO_2_ emissions are only a proxy for changes in atmospheric environment on account of aerosol concentration and size distribution at any particular time depending upon factors including coemissions and local meteorology, e.g., refs. 20 and 21. Detrended British SO_2_ emissions from 1800 to 1850, spanning Turner’s artistic production, correlate with detrended black carbon (r = 0.96) and organic carbon emissions (r = 0.95), indicating that variability in SO_2_ also generally tracks variability in other aerosol emissions and, thus, total aerosol concentrations. Later in the 19th century, however, the estimated emissions of black carbon and organic carbon per unit coal in England begin to decline (22). In London, in particular, political efforts to reduce industrial pollution (11), shifts in cooking and heating sources from coal to gas (18), and a more distributed urban landscape (9) that was enabled by an expanded railway network also likely contribute to decreasing peak aerosol concentrations (10, 18). We thus expect the magnitude of aerosol concentration associated with a given SO_2_ emission rate to decrease over the course of the 19th century.

## Trends in Contrast in Paintings by Turner, Monet, and Others

We examine the contrast of 60 oil paintings by Turner spanning 1796 to 1850 and 38 paintings by Monet spanning 1864 to 1901. Across Turner’s works (cataloged in *SI Appendix*, Fig. S4), a progression is visually apparent from sharp to hazier contours, more saturated to pastel-like coloration, and figurative to impressionistic representation. A similar progression is evident across Monet’s works (*SI Appendix*, Fig. S5), with the additional factor that Monet’s paintings are from two distinct locations. The first 18 of Monet’s paintings, dating from 1864 to 1872, depict scenes in or near Paris, and all but one were painted before Monet’s first visit to London from 1870 to 1871. The latter 20 paintings are from Monet’s 1899 to 1901 visits to London, where he created serialized views of the House of Parliament, Waterloo Bridge, and Charing Cross Bridge.

A mixed-effects model is used to evaluate whether local SO_2_ emissions contribute to variations in contrast across our collection of Turner and Monet paintings. In our baseline formulation, we specify fixed effects that capture variations in contrast according to SO_2_ emissions, year, and subject matter categories. We also allow for an interaction between year and SO_2_ to account for coemissions involved in producing atmospheric haze proportionately declining over time (22). Finally, the 98 paintings in our collections are partitioned into three categories, with 20 clear-sky, 46 cloudy, and 32 dawn or dusk paintings (*Methods*).

**Fig. 3.**
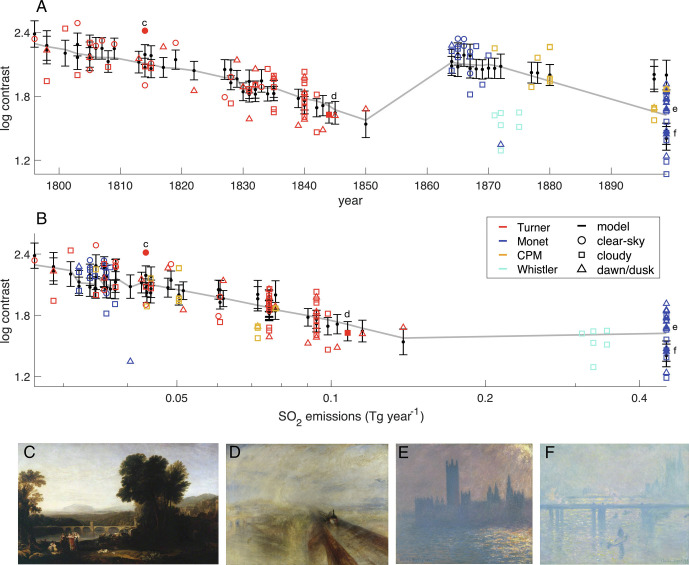
Trends in the contrast index for different subject matter in the 60 Turner paintings (red) and 38 Monet paintings (blue) versus (*A*) year or (*B*) SO_2_ emissions local to London or Paris. Also shown are six Whistler *Nocturnes* paintings (cyan), seven paintings by Caillebotte, four by Pissarro, and one by Morisot (gold). Paintings are categorized according to depicting conditions that are predominantly clear-sky (circle), cloudy (square), and dawn or dusk (triangle). Model predictions (black horizontal lines) are shown along with their 5 to 95% uncertainty (black vertical bars). Trends (gray lines) are illustrated by allowing year and SO_2_ to vary but withholding categorical fixed effect. Monet’s London paintings are plotted using 1899 London emissions because paintings were begun in the winter of 1899 to 1900, although exhibited in the following years, up until 1904. SO_2_ is plotted on a logarithmic scale. Also shown are four representative paintings: (*C*) Turner’s *Apullia in Search of Appullus* (1814), (*D*) Turner’s *Rain, Steam, and Speed* (1844), (*E*) Monet’s *Houses of Parliament, Sunlight Effect* (1899, in the Brooklyn Museum), and (*F*) Monet’s *Charing Cross Bridge* (1899, in Madrid’s Thyssen-Bornemisza Museum), with their values also highlighted in (*A*) and (*B*), as solid markers labeled with their panel letter.

Our baseline model explains 61% of the variance in the contrast index (Fig. 3, *SI Appendix*, Table S1). As expected, paintings depicting dawn or dusk conditions or cloudy conditions have a lower contrast index (*P* <  0.01) relative to clear-sky conditions. Moreover, the model shows a significant reduction in contrast in response to increases in SO_2_ emissions (*P* <  0.01), whereas the trend across years is indistinguishable from zero. The interaction effect is also significant (*P* <  0.01) and is consistent with the emissions of SO_2_ later in time yielding less change in the contrast index.

Six other model specifications are also explored that indicate that the significance of the SO_2_ contribution is robust to excluding the year term or admitting for quadratic contributions from SO_2_, year, or both (*SI Appendix*, Table S1). Our baseline formulation is selected from among these models because it balances simplicity against the major features that we are concerned with capturing. A means of selecting between models is offered by the Bayesian information criteria (BIC), which measures model performance as the difference between a reward term for better predicting observations and a penalty term based on the number of parameters that serves to guard against overfitting. A lower BIC indicates a more apt model. Our baseline specification gives among the lowest BICs, though admitting for nonlinear dependencies on year and SO_2_ gives comparable values. The least apt models, according to BIC, result from excluding SO_2_.

The primary reason that SO_2_, as opposed to year, is inferred to control contrast relates to the fact that Paris and London have distinct SO_2_ emission histories (Fig. 2). The magnitude of SO_2_ emissions in London near the beginning of Turner’s career in 1796 is similar to the magnitude of the emissions near the beginning of Monet’s career in Paris in 1864. Monet’s early paintings in Paris have higher contrast than most of Turner’s works subsequent to the 1820s, despite coming later, such that no simple time trend can be fit across these collections (Fig. 3*A*). If examined in the context of SO_2_ emissions, however, the contrast of Monet’s early works overlaps with those of Turner’s, and the low contrast of Monet’s later works is in accord with the high emissions in London at the end of the 19th century (Fig. 3*B*).

Monet and Turner are among the most prolific and iconic artists whose work spans the industrial era, but paintings by other artists that depict cityscapes and atmospheric phenomena also align with our proposed model. Specifically, our model predicts the contrast found in seven paintings by Gustave Caillebotte (1848 to 1894), four paintings by Camille Pissarro (1830 to 1903), and one painting by Berthe Morisot (1841 to 1895) of Paris on the basis of year, local SO_2_ emissions, and subject matter (Fig. 3). The contrast indices calculated for six *Nocturnes* paintings by Whistler in London between 1871 and 1875 are also predicted by our model. Note that Whistler’s paintings in less-polluted environments—for example, *The Coast of Brittany* (1861) or *The Blue Wave Biarritz* (1862)—are associated with substantially greater contrast indices of 2.4 and 2.2, respectively. Refitting the mixed-effects model to our expanded dataset including works by Caillebotte, Pissarro, Morisot, and Whistler leads to conclusions that are consistent with our more limited analysis of only works by Turner and Monet (Fig. 3). The year trend inferred from our expanded analysis, however, appears significant only in the case where SO_2_ is entirely excluded (*SI Appendix*, Table S1), further supporting the importance of SO_2_.

## Trends in Intensity

As a complementary approach, it is also possible to analyze the intensity of images across our collection of works. Aerosols scatter visible light of all wavelengths into the line of sight (23) (Fig. 1), leading to a whiter tint and increased light intensity during daytime (13). We examine the relationship between intensity and SO_2_ emissions using the same mixed-effects methodology used for contrast and find a significant effect (*P* <  0.01) of SO_2_ emissions increasing intensity in our baseline approach (*SI Appendix*, Table S2 and Fig. S6). Of the 12 other specifications including SO_2_, 9 show significant effects (*P* <  0.05), including all those conditioned on the larger set of artists. For the paired photographic analysis (*SI Appendix*, Fig. S2), polluted photos have a uniformly increased intensity index, consistent with the analysis of contrast, but in this case, averaging 39% greater. The interpretation of intensity trends is complicated, however, in that variations in image intensity may result from accumulation of residue, fading of pigments, or photographic techniques (24), in addition to optical effects created by aerosols, such that we consider intensity secondary to contrast for purposes of indicating optical effects.

Visibility can be inferred from intensity using an empirical relationship (*Methods*). Our estimates indicate that before 1830, visibility in clear-sky and cloudy Turner paintings averaged 25 km, whereas it decreased to an average of 10 km after 1830. For early Monet paintings, visibility averages 24 km, and for Monet’s daytime paintings in London, visibility averages 6 km (*SI Appendix*, Fig. S7). In comparison, (25) estimated visibility using the furthest clearly visible feature in 35 of Monet’s *Charing Cross Bridge* paintings and found a mean of 1 km. They note that the *London Fog Inquiry* describes visibility in the winter of 1901 to 1902 as never being more than approximately 2 km. Differences could arise due to uncertainties in both methodologies— the imprecision of estimating visibility by eye for (25) and, in addition to the aforementioned issues with interpreting intensity, there are various limiting assumptions in our model of visibility (*Methods*).

## Style vs. Environment

It is clear that industrialization changed the environmental context in which painting occurred. Indeed, 19th century art critic John Ruskin wrote about Turner’s work that, “had the weather when I was young been such as it is now, no book such as ‘Modern Painters’ ever would or could have been written” (26). A primary question, however, is the degree to which trends toward decreased contrast and increased intensity represent physical, optical changes associated with a polluted atmosphere, as opposed to exerting an indirect influence on artistic style. Beyond the statistical results discussed earlier, two further considerations suggest that environmental trends are rendered in the works we consider.

First, the environment that these artists depict was, in fact, subject to large trends in atmospheric pollution (17). Turner was born in the age of sail and died in an age of coal and steam (27). It is important to recognize, however, that not all artists depict a changed atmospheric environment. For example, John Constable (1776 to 1837) created works that show neither the diminished contrast nor increased intensity expected from London’s aerosol-laden atmosphere. It may be that certain artists chose times and locations where the effects of pollution were minimal. Indeed, while Constable remarked that Turner seems to paint with “tinted steam” (28), he himself was known to leave London for less-polluted Hampstead Heath or the Lake District (29).

The second consideration is more speculative as it relates to the intention of Turner and Monet to depict environmental change. We focus on Turner in this section and Monet in the next. Turner spoke about finding artistic material in his environment: “nature dispensing incidents for the artist’s study... to store in his mind with every change of time and place” (30). More specifically, Turner sought to represent technological and resulting environmental change (27), especially as it relates to atmospheric effects on light. In *The Fighting Temeraire* (1839), perhaps Turner’s most iconic work, a steam-powered tugboat pulls the HMS Temeraire, a military sailing ship made famous by the 1805 Battle of Trafalgar, to land to be broken up for scrap against a backdrop of a fiery setting sun, illustrating the transition from the age of sail to steam. Similarly, *Rain, Steam, and Speed* (1844) depicts a train racing through the British countryside, contrasted with symbols of the past age, such as a row boat gliding over the water, a hare, the fastest natural animal in Britain, running from the oncoming train, and a farmer plowing without mechanized equipment, all almost lost in mist.

That Turner should be among the first to depict changes in how light transmits through a polluted atmosphere might be traced to a general increase in the interest in and scientific understanding of light and the sky that occurred during his lifetime (31). In 1801, astronomer William Herschel gave a lecture, “The Nature of the sun” (32), which is thought to have influenced how Turner paints the brightness and texture of the Sun (27) (*SI Appendix*, Fig. S8*A*). In 1803, meteorologist Luke Howard published *On the Modification of Clouds* that introduced the cloud classification of cumulus, stratus, and cirrus (33), which was featured in art manuals and even inspired a poem by Johann Wolfgang von Goethe (1749 to 1832) (29). *SI Appendix*, Fig. S8*B* shows cloud studies by Luke Howard, and, roughly synchronously, by Turner.

Turner’s documentation of the optical effects of aerosols is also on display in the context of explosive volcanic eruptions. Turner’s paintings show changes in sunset coloration that accord with the expected effects of volcanic eruptions injecting aerosols into the stratosphere (5, 6). Turner also produced a sketchbook of 65 watercolors of sunsets in the three years following the Tambora eruption that captures the waxing and waning of the atmospheric reddening associated with stratospheric volcanic aerosols (*SI Appendix*, Fig. S8*C*). The fact that the course of events that Turner documents is consistent with the expected timescale associated with stratospheric aerosol migration and deposition following a volcanic eruption, (i.e., 1 to 3 y, 34) is further evidence for Turner providing a faithful depiction of variations in atmospheric light phenomena.

## Additional Considerations for Inferring Pollution from Paintings

If it is accepted that the optical consequences of increased atmospheric pollution are depicted in certain works, a question arises whether it is possible to calibrate the depicted trends for making inferences regarding atmospheric composition. Although such a reconstruction would be useful because there are no direct quantitative measurements of urban air pollution during early industrialization (35), any such inference is challenging. The mechanisms associated with recording environmental conditions using paint and canvas are, arguably, of similar complexity to how any natural proxy records the environment, such as tree rings or ice cores. The aesthetic considerations associated with works of art then add additional layers of interpretation. One issue is that Monet and Whistler appear to have been influenced by Turner’s style (36). Turner’s *Rain, Steam, and Speed*, for example, was one of the few paintings by other artists that Monet directly referred to in his correspondence (37).

A related issue in considering whether atmospheric composition can be inferred from certain paintings is that the scenes sampled in these works are not chosen at random. Monet, for example, wrote about the role of air pollution in his creative process, “What I like most of all in London is the fog” (11) and, “when I got up I was terrified to see that there was no fog, not even a wisp of mist: I was prostrate, and could just see all my paintings done for, but gradually the fires were lit and the smoke and haze came back” (38). Note that the word “smog” for smoke and fog was not coined until 1905 (11). Insomuch as Monet focused on the atmospheric effects associated with high aerosol concentrations, trends in painting characteristics may reflect changes in extreme events, as opposed to reflecting changes in average conditions.

There is some evidence that Monet chose to paint on days when ambient air pollution would have been higher on account of meteorological conditions. Given some amount of aerosol precursor emissions, ambient air pollution concentrations tend to be higher when surface winds are weak, surface pressure is high, and precipitation is absent (39, 40). This meteorological pattern was already speculated upon in the context of “London Fogs” around the time that Monet was painting (41). Monet’s letters indicate that he painted on 26 February and 4, 7, and 9 March 1900 (38), and the corresponding daily weather reports for London (42) indicate that these days are associated with weaker wind speeds (varying from 1 to 3 out of 12 in the Beaufort wind scale), relatively higher atmospheric pressure (with phrases like “barometer rising” or “bar rising slowly”), and essentially no precipitation (noted as 0 inches on these days, except 0.01 inches on 26 February). It is also well established that air pollution concentrations are higher in winter than summer because of a shallower planetary boundary layer and because atmospheric stability can be higher on account of capping inversions (43), consistent with Monet visiting and painting London in winter and spring months. Information is lacking, however, regarding the specific time, date, or dates individual paintings depict, such that we do not explicitly account for meteorological reports or seasonal dependencies in our analysis.

We also mention a hypothesis that ascribes trends in works to increasingly faulty vision (44). It appears that loss of visual acuity associated with the development of cataracts led Edgar Degas (1834 to 1917) to paint with a different palette and in less detail (45). There is no direct evidence, however, that Turner, or Monet for that matter, had eyesight conditions that would translate into hazier paintings styles (46). Turner continued to paint details in the foreground throughout his life, and (46) demonstrates that Monet was not myopic and that he suffered from cataracts decades after he began painting more impressionistic works.

## Conclusions

Our basic premise is that Impressionism—as developed in the works of Turner, Monet, and others—contains elements of polluted realism. Over the 19th century, the atmospheric reality in London and Paris changed. Turner, Monet, and others document these changes in paint, yielding proxy evidence for historical trends in atmospheric pollution before instrumental measurements of air pollution become available. A mixed-effects model including both temporal and environmental trends can explain 61% of the variance in a contrast index and gives a significant dependence on SO_2_ emissions for each statistical model specification, including after controlling for year and subject matter. These results indicate that a combination of trends in style and atmospheric pollution contribute to trends in the contrast of Turner and Monet’s paintings. The magnitude of the changes in paintings is plausible relative to changes between contemporary pairs of clear-sky and polluted photographs. Estimates of intensity generally correspond to those of contrast but are noisier and less significant. Visibility inferred from the London works by Monet is also in keeping with historical records of visibility.

Issues associated with scene selection and the atmospheric chemistry of smog would need to be controlled for before quantitative inferences of mean atmospheric conditions are possible from this sample of paintings. Nevertheless, the evidence that we present for Turner, Monet, and others depicting physical atmospheric conditions provides additional, complementary opportunities for appreciating and interpreting their artwork. Our view is that impressionistic paintings recording natural phenomena—as opposed to being imagined, amalgamated, or abstracted—does not diminish their significance; rather, it highlights the connection between environment and art. Furthermore, our results suggest that environmental change provided a creative impulse whereby the importance of lines and edges was diminished in favor of demarcating objects using color fields.

A historical connection between aerosols and painting style may also afford some perspective on cultural responses to contemporary human-caused environmental changes. Megacities such as Beijing, New Delhi, and Mexico City have levels of air pollution similar to those of 19th century London (47). Furthermore, if stratospheric solar radiation management were used to mitigate climate risk (48, 49), it would increase the intensity, or whiteness, of the sky and globally diminish the contrast of objects viewed against this background. Our findings suggest that modern changes to atmospheric properties can also be expected to both literally and figuratively change how we see the world.

## Materials and Methods

### Theoretical Expectations of Contrast and Visibility

As the distance, *dx*, between an observer and object increases, the intensity of light from the object, *I*_o_(*x*), increases as a result of diffuse background light scattered into the line of sight, *σ*_b_*I*_b_(*x*), and decreases as a result of scattering and absorption along the line of sight, *σ*_e_*I*_o_(*x*),

[2] $$\begin{array}{c} \frac{dI_{o}\left(x\right)}{\mathrm{dx}}=\sigma_{b}I_{b}\left(x\right)-\sigma_{e}I_{o}\left(x\right). \end{array}$$

When particles are present, *dx* is proportional to the number of suspended aerosol particles in the air column. The isotropic scattering coefficient, *σ*_b_, represents the efficiency with which background light is scattered into the line of sight, and the extinction coefficient, *σ*_e_, represents how much intensity is lost through absorption and scattering as a beam of light passes through a material. Both coefficients are in units of inverse meters.

Unlike for a finite object, background radiation is assumed to be independent of *x*, given homogeneous, isotropic background scattering, and therefore,

[3] $$\begin{array}{c} \frac{dI_{b}\left(x\right)}{\mathrm{dx}}=\sigma_{b}I_{b}\left(x\right)-\sigma_{e}I_{b}\left(x\right)=0. \end{array}$$

It follows that *σ*_b_ equals *σ*_e_.

Replacing *σ*_b_ with *σ*_e_ in Eq. **2**, integrating intensity from 0 to *I* and distance from 0 to *X*, and taking the logarithm yields

[4] $$\begin{array}{c} \frac{I_{b}\left(x\right)-I_{o}\left(x\right)}{I_{b}\left(x\right)}=\exp\left(-\sigma_{e}X\right). \end{array}$$

The left-hand side of Eq. **4** is defined as the contrast, *C*(*x*), or the relative difference between *I*_b_(*x*) and *I*_o_(*x*),

[5] $$\begin{array}{c} C\left(x\right)=\exp\left(-\sigma_{e}X\right). \end{array}$$

A black object at a distance *x* = 0, for instance, has *I*_o_(0)=0, by definition, yielding a contrast of one. Eq. **4** is a version of the Beer–Lambert law where contrast decreases exponentially with distance from an object, *dx*, or with particle concentration when particles are present. Assumed in this representation is that both the background and object intensities are seen along nearly the same lines of sight and that the background intensity is independent of direction (12).

Taking the logarithm of Eq. **5** and rearranging yields,

[6] $$\begin{array}{c} X=\frac{-\ln(C(x))}{\sigma_{e}}. \end{array}$$

Earlier studies assumed that a contrast threshold of *C*(*x*)=0.02 was the perceptible limit or farthest distance one can detect a dark object against a light background (50). We follow more-recent studies in using a contrast threshold of 0.05 (51, 52), yielding a highly idealized estimate of visibility, *X*_v_, as an inverse function of the extinction coefficient, *σ*_e_,

[7] $$\begin{array}{c} X_{v}=\frac{3.0}{\sigma_{e}}, \end{array}$$

known as the Koschmieder equation (50).

#### Intensity and Visibility.

We quantify the amount of white light using the hue–saturation–intensity color model, where intensity ranges from black, with a value of zero, to white with a value of one (53). We consider the median intensity across all image pixels, referred to as the intensity index. The image median is simple to define, though an analysis of only the sky or other common features could also be instructive.

It is possible to estimate *σ*_e_ from anomalies in intensity using an empirical function derived from photographic observations (13),

[8] $$\begin{array}{c} \sigma_{e}=3.4\times 10^{-7}\exp\left(14.7(\overset{¯}{I}-{\overset{¯}{I}}_{95})\right)+1.1\times 10^{-4}. \end{array}$$

$\overset{¯}{I}$ is the image-median intensity, which ranges from 0.1 to 0.8 across the images of paintings that we consider (*SI Appendix*, Fig. S6) and between 0.3 to 0.9 among the urban photographs we consider (*SI Appendix*, Fig. S2). Depending on the application of Eq. **8**, ${\overset{¯}{I}}_{95}$ is the 95th percentile of the clear-sky paintings in our collection, 0.65, or the 95th percentile of clear-sky photographs in our collection, 0.75. Note that Eq. **8** is rewritten from (13) to be in units of inverse meters and to depend on intensity scaled between 0 and 1.

A less-idealized estimate of *σ*_e_ would be possible taking into account solar geometry, the position of the observer, and the direction of view, but such information is not readily available for most of the paintings we consider. The images we consider, moreover, are not digitized under identical conditions, which inevitably introduces noise to the samples.

Substituting Eq. **8** for *σ*_e_ into Eq. **7** gives an estimate of the visibility range associated with various paintings (*SI Appendix*, Fig. S7). For early Turner works, our visibility ranges are broadly consistent with ranges of 20 to 30 km for contemporary clear-sky urban conditions (54), and for Turner’s later works, as well as Monet’s London paintings, visibility ranges are consistent with the 1 to 5 km range estimated for contemporary strong urban haze conditions (13). Visibility estimates below 5 km for late 19th century London are in keeping with estimates for contemporary megacities during strong urban haze conditions, such as Delhi (55), and Beijing (56, 57).

Fractional changes in contrast and intensity indices are also calculated for comparison with photographs. This percent change is calculated only for Turner paintings (e.g., by subject matter, such as predominantly clear-sky) by dividing these paintings into two groups and computing, (*I*_late_ − *I*_early_)/*I*_early_ × 100, wherein paintings in each category are divided into two equally sized groups for early and late. For photographs, the percent change is calculated between clear-sky and polluted photographs.

#### Wavelet Analysis of Contrast.

Wavelet analysis is performed by convolving grayscale image matrices with a two-dimensional, multiscale Haar wavelet using the Python package, *PyWavelets* and, specifically, the *wavedec2* function (58). The Haar wavelet consists of a hierarchy of square-wave–shaped functions,

[9] $$\psi (t)=\{\begin{array}{ll} 1 & 0\leq t<\frac{1}{2},\\ -1 & \frac{1}{2}\leq t<1,\\ 0 & \text{otherwise}, \end{array}$$

Grayscale image matrices are calculated as a weighted sum of the corresponding red, green, and blue pixels, X = 0.2125R + 0.7154G + 0.0721B, though results are qualitatively similar for individual color channels.

High-pass or detailed coefficients are interpreted because of interest in the representation of abrupt features. Seven scales are used for the Haar wavelets, although similar results are obtained using fewer scales. Coefficients can be computed in the horizontal, vertical, and diagonal directions, and we use horizontal coefficients that emphasize horizontal edges (59).

An index of the contrast found in an image is computed as the 95th-percentile of all high-pass horizontal coefficients divided by the median (Eq. **1** in *Optical Implications of Increasing Aerosol Concentrations*). Selection of the 95th-percentile represents a balance between identifying among the sharpest features in an image and guarding against being overly sensitive to outliers. Using other high percentiles, however, such as the 90th or 99th, gives similar results. Normalization by the median of the coefficients accounts for different baseline edge strengths depending on lighting, scene, and image resolution.

## Mixed-Effects Model Formulation

Our baseline mixed-effects model, for the contrast index is formulated as follows:

[10] $$\begin{array}{c} \log(\mathrm{contrast})=\alpha_{0}\;+\;\alpha_{1}\mathrm{year}\;+\;\alpha_{2}{\mathrm{SO}}_{2}\;+\;\alpha_{3}\left(\mathrm{type}\right)\;+\;\alpha_{4}\mathrm{year}*{\mathrm{SO}}_{2}\;+\;\epsilon_{c}. \end{array}$$

See ref. (60) for an overview of mixed-effects models. This model involves fixed effect terms for year, SO_2_, the interaction between year and SO_2_, and categorical effects associated with subject matter (type) according to clear-sky, cloudy, or dawn/dusk conditions. Allowing for random intercepts or slopes, such as for type, does not improve the fit as judged by either the Akaike or Bayesian information criteria, consistent with our prior expectation of a nonzero contribution to contrast from each factor. The year is represented as the anomaly from the mean. The interaction term between year and SO_2_ captures the weakening effect of a given amount of SO_2_ to the total aerosol concentration (*Quantifying Historical Air Pollution*). This interaction term is positive and offsets the negative contribution to contrast solely from SO_2_, which overpredicts decreases in contrast when SO_2_ is considered individually, without the interaction term.

This model formulation allows for examining how SO_2_ emissions influence contrast across paintings after controlling for effects associated with temporal trends and selection of subject matter. Although year and SO_2_ are correlated (R^2^ = 0.47), the fact that London and Paris have different SO_2_ time histories permits for distinguishing simple time trends from environmental trends.

Results for our primary specifications are given in *SI Appendix*, Table S1 including only works by Turner (60) and Monet (38) on row 1 and for also including 6 works by Whistler, 7 by Caillebotte, 4 by Pissarro, and 1 by Morisot on row 7. Six alternative specifications are also examined: omitting the interaction term between year and SO_2_, omitting the interaction and SO_2_ terms, omitting the interaction and year terms, omitting the interaction term but including a term for SO_2_^2^, omitting the interaction term but including year ^2^, and omitting the interaction term but including both year ^2^ and SO_2_^2^. Specifications 8 to 14 are equivalent to 1 to 7 but applied to the larger collection of 116 paintings.

Similarly, a linear model for the intensity index is formulated as follows:

[11] $$\begin{array}{c} \mathrm{intensity}=\beta_{0}+\beta_{1}\mathrm{year}+\beta_{2}{\mathrm{SO}}_{2}+\beta_{3}\left(\mathrm{type}\right)+\beta_{4}\mathrm{year}*{\mathrm{SO}}_{2}+\epsilon_{i}. \end{array}$$

*SI Appendix*, Table S2 reports intensity results for the baseline formulations for Turner and Monet paintings (row 1) and for all paintings (row 7) as well as six alternative specifications as for contrast (14 specifications total; seven for Turner and Monet paintings and another seven the larger collection of paintings).

## Supplementary Material

Appendix 01 (PDF)

## Data Availability

Painting, air pollution data have been deposited in Harvard Dataverse (https://doi.org/10.7910/DVN/YQOLZW). All study data are shown in the article and/or *SI Appendix*.
