# Supplementary material for: Paintings by Turner and Monet depict trends in 19th century air pollution
Source: Proc Natl Acad Sci U S A. 2023 Jan 31;120(6):e2219118120. doi: 10.1073/pnas.2219118120 (PMC9963082; doi:10.1073/pnas.2219118120)
Supplement: Supplementary file 1 — Appendix 01 (PDF) [file pnas.2219118120.sapp.pdf]

Supporting Information for

Paintings by Turner and Monet Depict Trends in 19th Century Air Pollution

Anna Lea Albright, Peter Huybers

Anna Lea Albright. Email: anna-lea.albright@lmd.ipsl.fr

This PDF file includes:

Figs. S1 to S8
Tables S1 to S2

	R^2	BIC	year	SO_2	dawn	cloudy	year*SO_2
1.	0.61	-25.1	-0.46** (-0.69 -0.23)	-5.42** (-6.86 -3.99)	-0.09	-0.12*	0.09**
2.	0.43	6.6	-0.04 (-0.27 0.19)	-0.81** (-1.29 -0.34)	-0.21**	-0.23**	-
3.	0.36	13.1	-0.35** (-0.50 -0.20)	-	-0.27**	-0.24**	-
4.	0.43	2.2	-	-0.89** (-1.18 -0.59)	-0.22**	-0.23**	-
5.	0.61	-26.1	-0.14 (-0.34 0.05)	-8.19** (-10.40 -5.98)	-0.08	-0.11*	-
6.	0.57	-17.2	0.33** (0.09 0.56)	-2.32** (-2.98 -1.66)	-0.12	-0.14**	-
7.	0.61	-22.5	-0.36 (-0.84 0.12)	-10.44** (-15.47 -5.42)	-0.08	-0.11*	-
8.	0.60	-28.2	-0.52** (-0.73 -0.32)	-4.85** (-6.08 -3.61)	-0.10	-0.11*	0.09**
9.	0.45	4.7	-0.04 (-0.21 0.13)	-0.84** (-1.21 -0.46)	-0.22**	-0.21**	-
10.	0.36	17.8	-0.29** (-0.44 -0.15)	-	-0.28**	-0.21**	-
11.	0.45	0.1	-	-0.89** (-1.18 -0.61)	-0.22**	-0.22**	-
12.	0.63	-35.5	-0.12 (-0.27 0.03)	-8.62** (-10.72 -6.51)	-0.08	-0.10	-
13.	0.50	-1.3	0.08 (-0.10 0.26)	-1.27** (-1.72 -0.83)	-0.19**	-0.18**	-
14.	0.63	-31.3	-0.16 (-0.33 0.02)	-9.11** (-11.58 -6.64)	-0.07	-0.10	-

Table S1. Summary statistics for mixed effects models of log contrast. For each of 14 specifications we report variance explained by each model (R^2), the Bayesian Information Criteria (BIC), and the fixed effects coefficients for year (contrast index units per century), SO_2 (contrast index units per Tg SO_2 emitted per year), interaction between year and SO_2, and categorical offsets from a clear-sky baseline for paintings depicting dawn/dusk or cloudy conditions (contrast index units). The 95% confidence interval for fixed effects associated with year and SO_2 are reported parenthetically. Coefficients that differ from 0 at $p < 0.05$ are indicated by '*' and by $p < 0.01$ by ''. The baseline specification, no. 1, is for the contrast index in the 98 Turner and Monet paintings to equal $\alpha_0 + \alpha_1$year $+ \alpha_2 SO_2 + \alpha_3(\text{type}) + +\alpha_4$year*$SO_2 + \epsilon_c$, wherein $\alpha_3(\text{type})$ is expanded as $\alpha_{\text{dusk}} + \alpha_{\text{cloud}}$. Specification 2 omits the interaction term between year and SO_2; 3 omits the interaction and SO_2 terms; 4 omits the interaction and year terms; 5 omits the interaction term but includes a term for SO_2^2; 6 omits the interaction term but includes year^2; and 7 omits the interaction term but includes both year^2 and SO_2^2. Specifications 8–14 are equivalent to 1–7 but applied to a collection of 116 paintings that include works by Whistler, Caillebotte, Pissarro, and Morisot.**

	R^2	BIC	year	SO_2	dawn	cloudy	year*SO_2
1.	0.37	-90.4	0.36** (0.20 0.53)	2.32** (1.29 3.35)	0.04	0.07	-0.04**
2.	0.23	-75.3	0.15* (0.00 0.31)	-0.01 (-0.32 0.31)	0.10*	0.13**	-
3.	0.23	-79.9	0.15** (0.06 0.25)	-	0.10*	0.13**	-
4.	0.19	-75.9	-	0.24* (0.04 0.44)	0.10*	0.14**	-
5.	0.36	-89.2	0.20** (0.06 0.34)	3.57** (1.97 5.17)	0.04	0.07	-
6.	0.38	-92.8	-0.06 (-0.22 0.10)	0.88** (0.43 1.33)	0.05	0.08*	-
7.	0.38	-88.2	-0.10 (-0.44 0.24)	0.46 (-3.14 4.05)	0.05	0.08*	-
8.	0.35	-103.6	0.38** (0.23 0.53)	2.09** (1.20 2.98)	0.04	0.07	-0.04**
9.	0.22	-88.0	0.13* (0.01 0.25)	0.04 (-0.22 0.29)	0.10*	0.12**	-
10.	0.22	-92.7	0.14** (0.05 0.23)	-	0.10*	0.12**	-
11.	0.19	-88.0	-	0.22* (0.02 0.42)	0.11*	0.14**	-
12.	0.35	-103.7	0.17** (0.06 0.28)	3.74** (2.17 5.31)	0.03	0.07	-
13.	0.30	-95.9	0.04 (-0.08 0.16)	0.36* (0.06 0.65)	0.08	0.10**	-
14.	0.35	-100.3	0.13 (-0.00 0.26)	3.17** (1.34 5.01)	0.04	0.07	-

Table S2. Summary statistics for mixed effects models of intensity. As for Table S1, but Whistler's *Nocturnes* paintings are excluded, as they depict dark, nighttime conditions that, by definition, have much lower intensity.

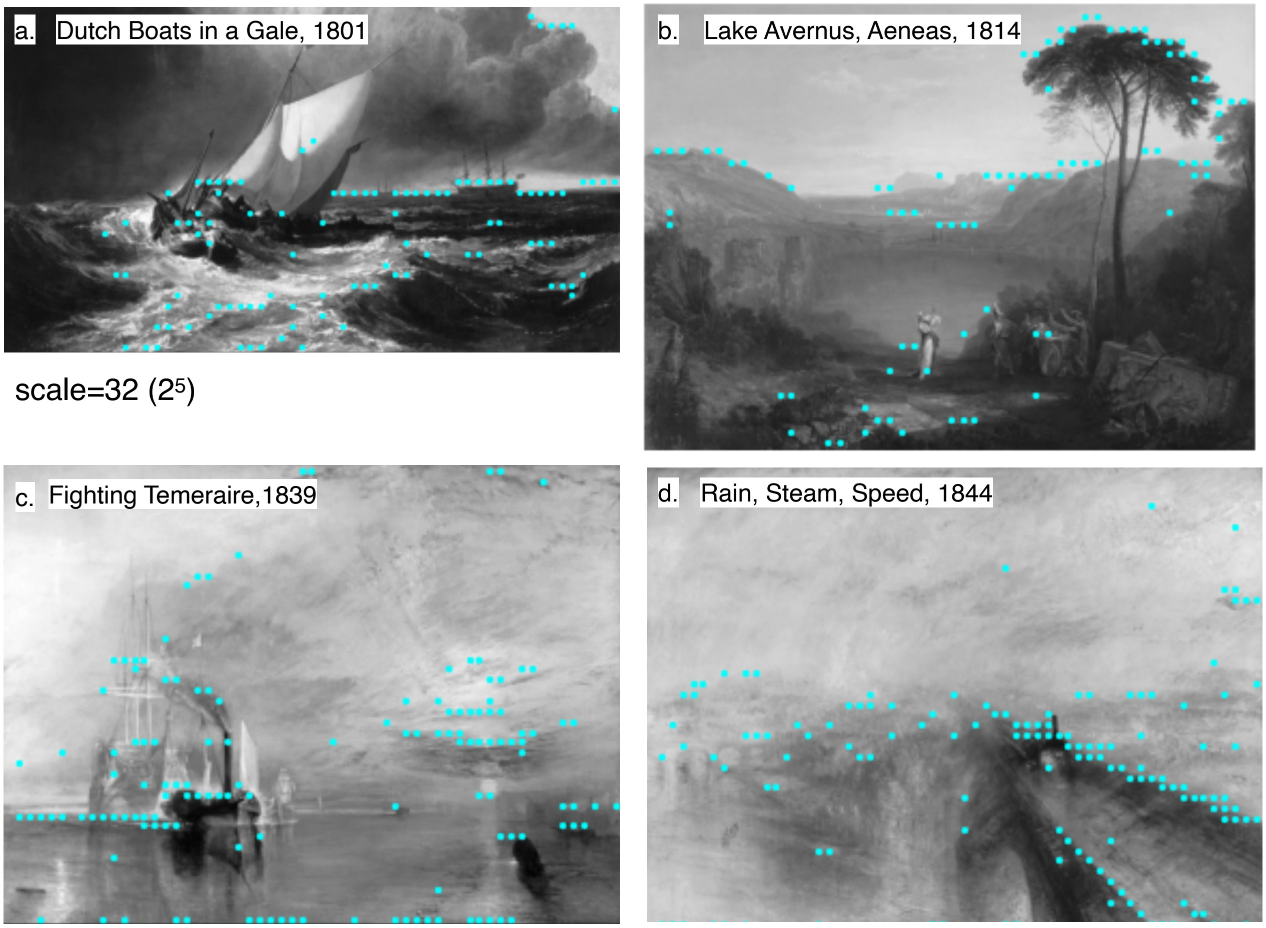

Fig. S1. Four example paintings from Turner from (a.) *Dutch Boats in a Gale* (1801); (b.) *Lake Avernus–Aeneas and the Cumaean Sybil* (1814); (c.) *The Fighting Temeraire* (1839); and (d.) *Rain, Steam, Speed* (1844). Horizontal coefficients at the fifth wavelet scale (i.e., the difference across $2^5 = 32$ pixels) that exceed the 95th-percentile are indicated in each painting with cyan dots.

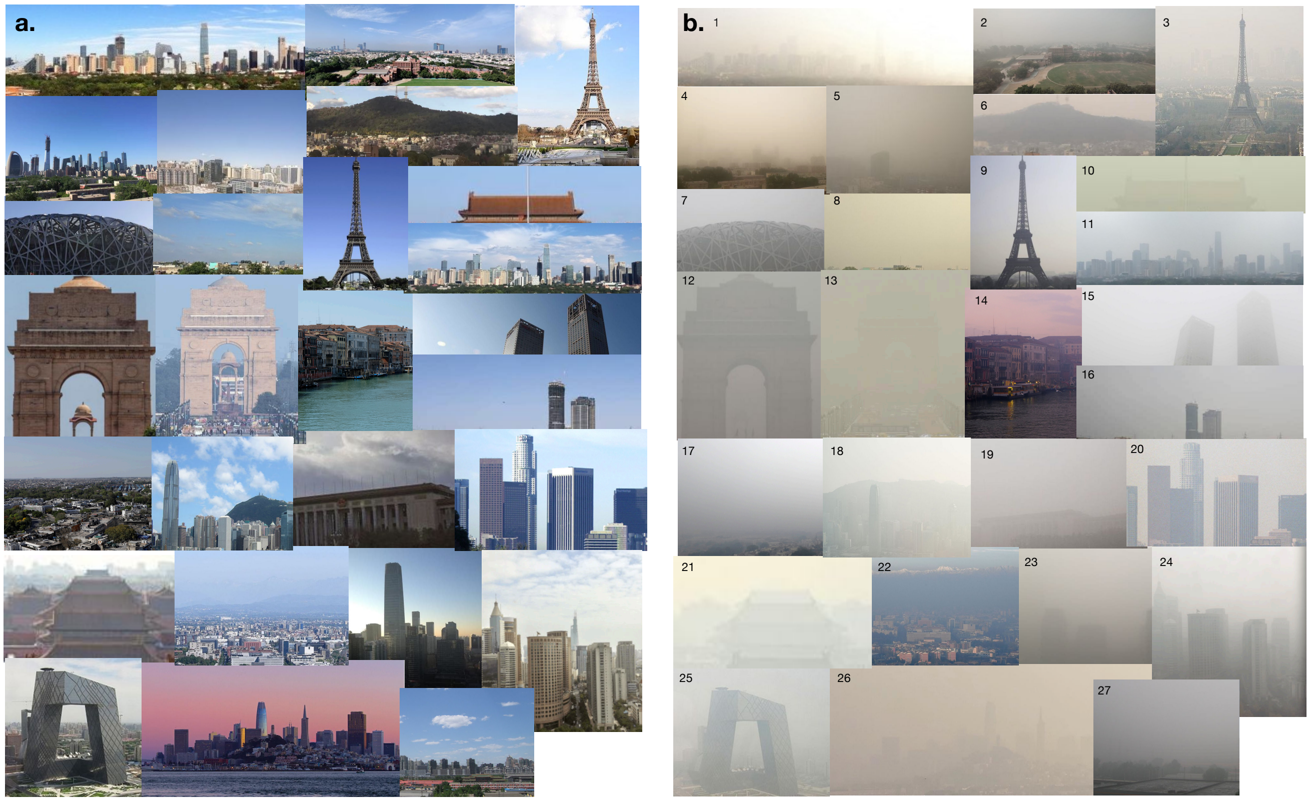

Fig. S2. 27 pairs of contemporary clear-sky (a.) and polluted (b.) photographs that depict the same scene but differ in the level of pollution. (c) Log-contrast values for clear-sky (blue) and polluted (grey) photographs, and (d) image-median intensity values for clear-sky and polluted photographs.

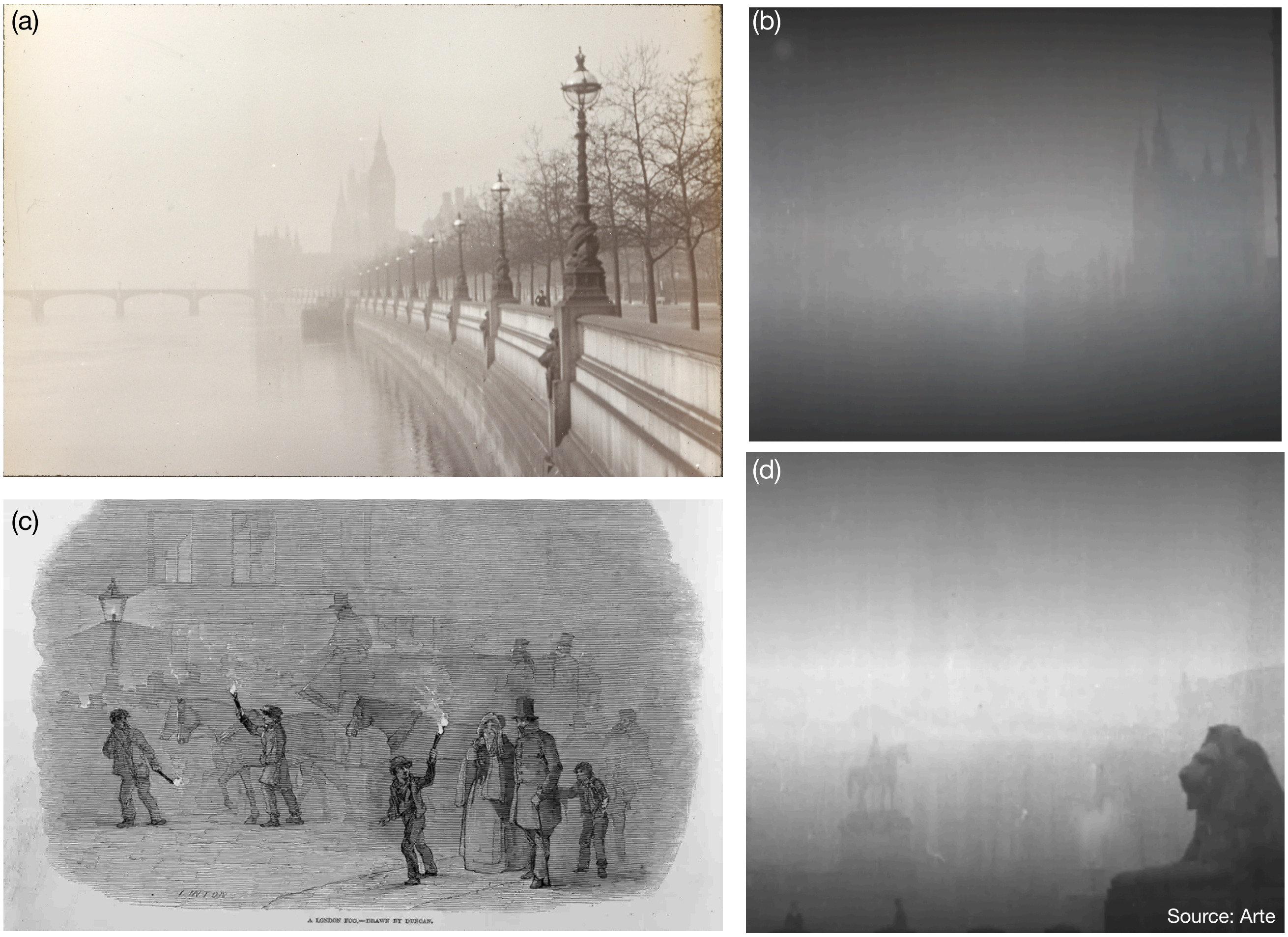

Fig. S3. Various illustrations of the optical effects associated with historical London air pollution. (a): ‘London. Embankment’ (1908) by British photographer Frederick Evans (1853–1943). Note that photographic emulsions used prior to about the 1920s were mostly sensitive to blue light and could not differentiate clouds from non-cloudy sky, which could perhaps emphasize the hazy, luminous impression of the photograph. Atmospheric effects can therefore not be directly inferred from the image. (b) and (d) Extracts from a documentary ‘Monet in the garden of Clemenceau’ on *Arte* by François Prodromidès depicting London smogs, though they are taken in the early 20th century instead of during our period of study. (c) A sketch in the Illustrated London News, Volume 10 from the year 1847, illustrating how pedestrians and carriages were guided with torches, as the thick fogs reduced visibility even across a street, and fogs could be a cover for thievery (see lower right corner). Londoners were, moreover, aware of the dangers of fogs for health, such as increased deaths from bronchitis and other respiratory diseases, as documented in contemporary sources (as reviewed, for instance, by Corton, 2015).

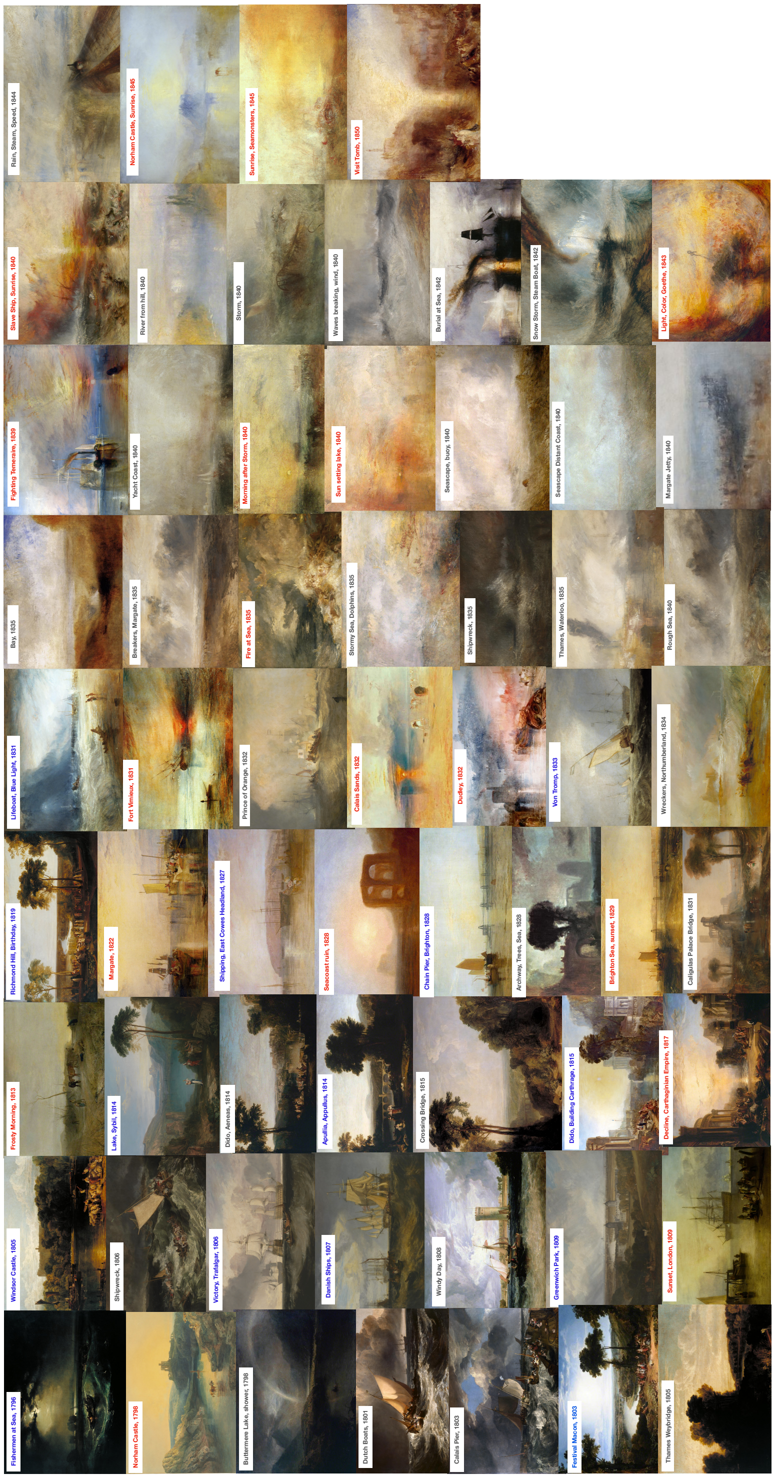

Fig. S4. 60 oil paintings by Turner from 1798–1850 analyzed in Fig. 3 and Fig. S6. Each painting is indicated by keywords and year. Time goes down the columns and left to right. Text colors indicate the category: predominantly clear-sky (blue), predominantly cloudy (grey), and dawn/dusk (red). Certain paintings are cropped in this image but none are cropped in the analysis.

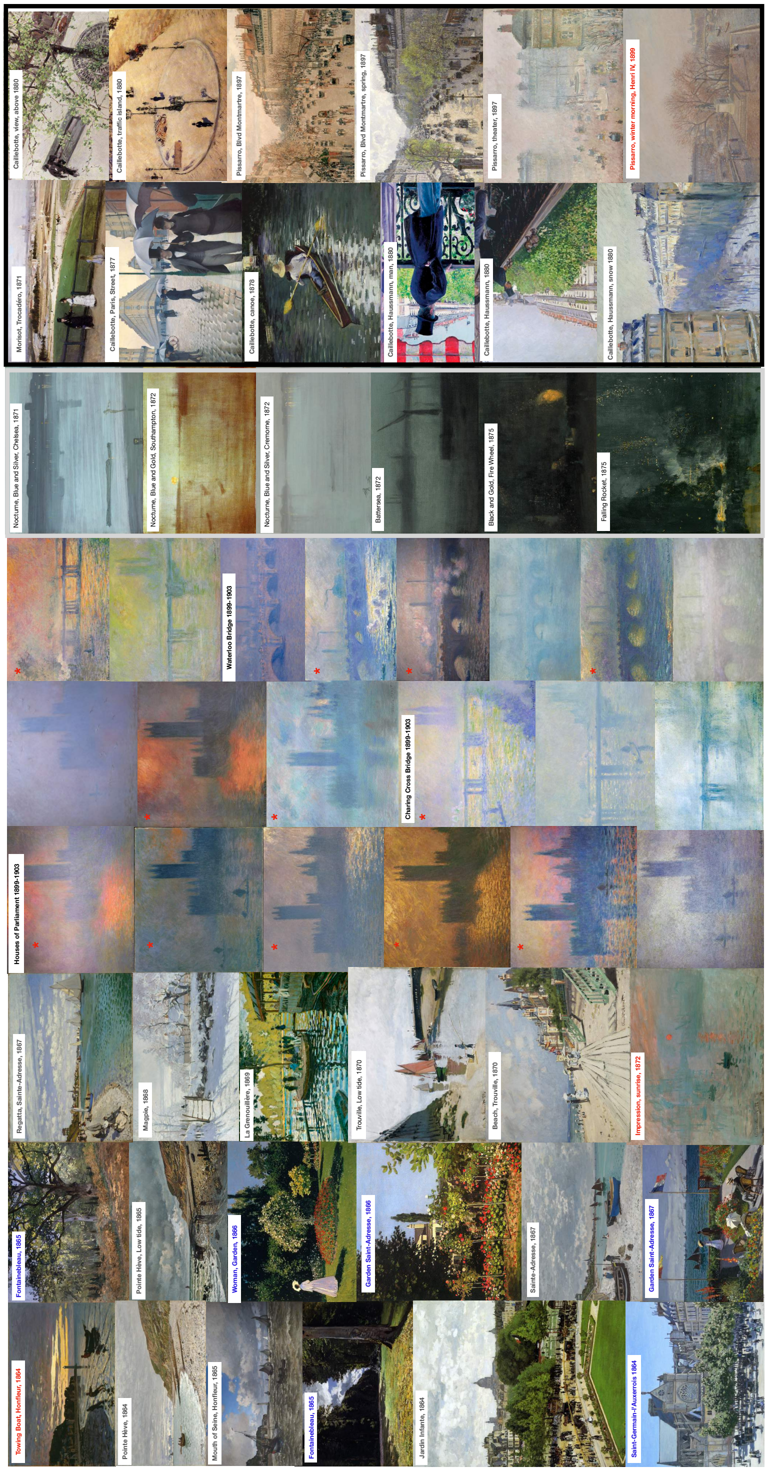

Fig. S5. 38 oil paintings by Monet from 1864–1904 used in this analysis along with six from Whistler. As for Turner, we include keywords identifying the painting and its year of creation. Time goes down the columns and left to right; and colors of these keywords and date indicate the category: predominantly clear-sky (blue), predominantly cloudy (grey), and dawn/dusk (red). For Monet's series paintings in London of dawn/dusk are indicated by a red star in the top left corner. The remaining paintings are classified as predominantly cloudy or hazy. Outlined in the grey box are six London *Nocturnes* by Whistler from 1871–1875. Outlined in black are seven paintings by Caillebotte, four by Pissarro, and one by Morisot.

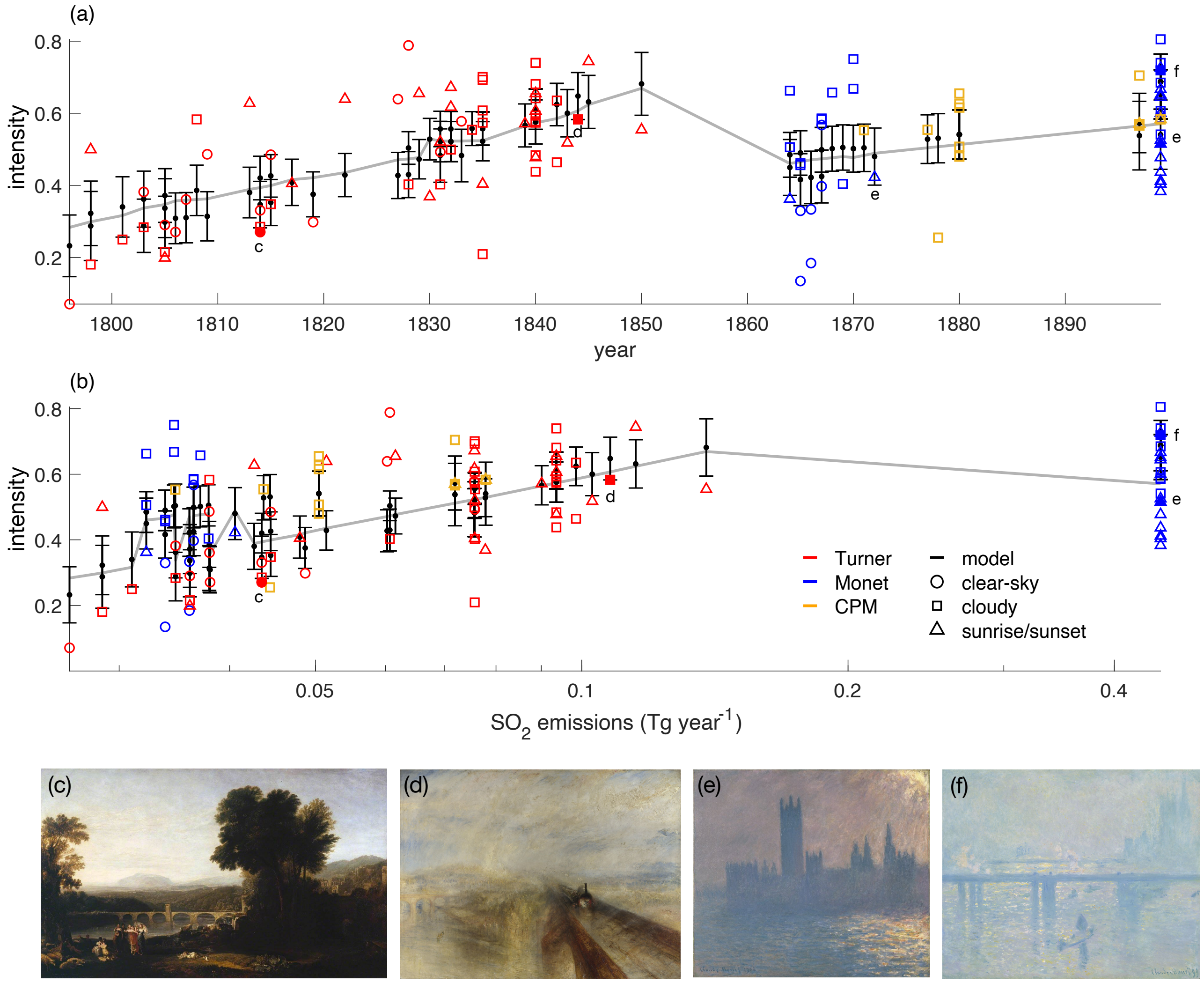

Fig. S6. Trends in the intensity index for different subjects in the 60 Turner paintings (red) and 38 Monet paintings (blue) versus (a.) year or (b.) respective London or Paris SO_2 emissions. Paintings by Caillebotte (7), Pissarro (4), and Morisot (1) are in gold. Marker styles correspond to painting type: predominantly clear-sky (circle), predominantly cloudy (square), and dawn/dusk (triangle). Black horizontal lines are model predictions, and black vertical bars indicate the 5–95% uncertainty on the model prediction. Note that year is normalized by its mean in the analysis but plotted without this normalization for reference; SO_2 is plotted on a logarithmic scale; and all Monet paintings are plotted using 1899 London emissions because paintings were begun in the winter of 1899–1900 but first exhibited in the following years, up until 1904. Also shown are four representative paintings: (c) Turner's *Apullia in Search of Appullus* (1814), (d) Turner's *Rain, Steam, and Speed* (1844), (e) Monet's *Houses of Parliament, Sunlight Effect* (1899, now in Brooklyn Museum), and (f) Monet's *Charing Cross Bridge* (1899, now in Madrid's Thyssen-Bornemisza Museum), with their values also highlighted in panels (a) and (b), as solid markers labelled with their panel letter.

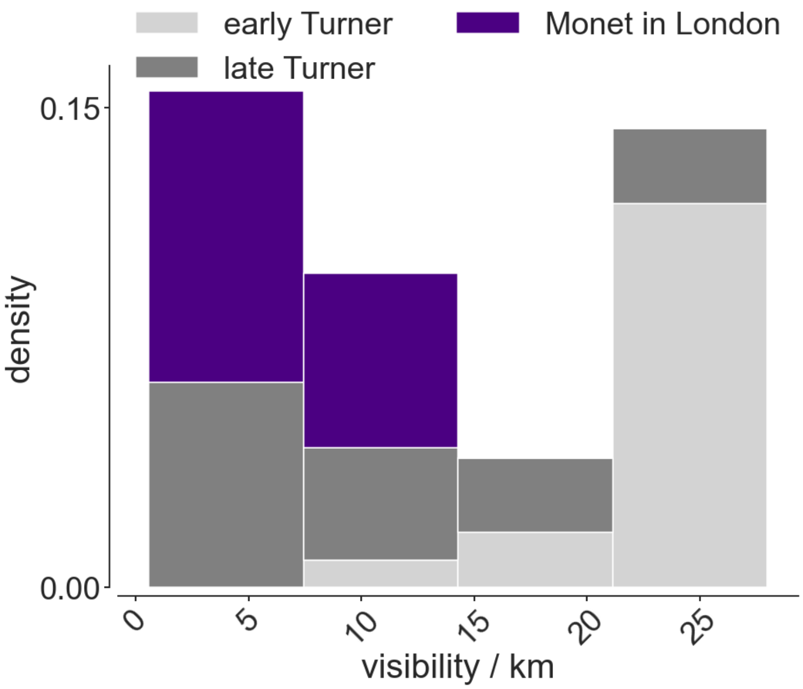

Fig. S7. Estimates of visibility using Eq. 6 for clear-sky and cloudy Turner paintings before 1830 (light grey) and after 1830 (dark grey), as well as daytime (excluding sunrise and sunset) Monet paintings in London (purple).

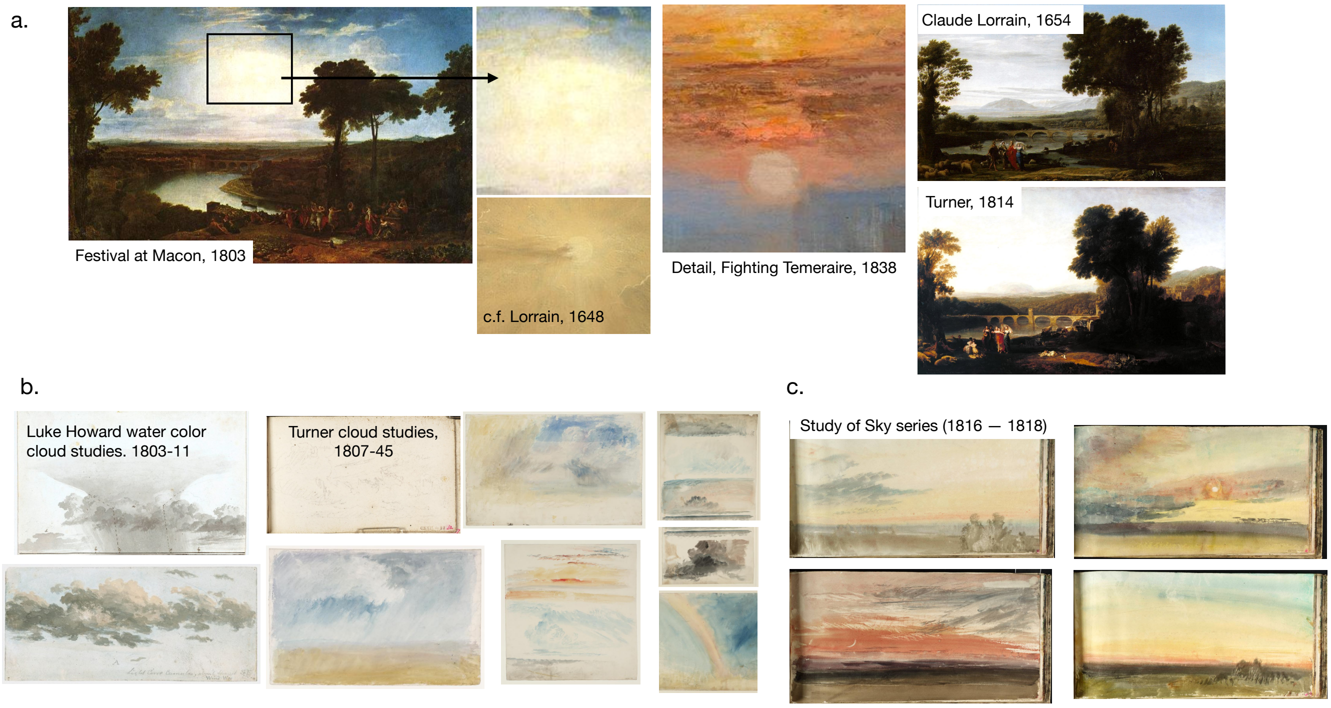

Fig. S8. Tracing Turner's awareness of growing scientific understanding of the changeability of the sun and the environment with three vignettes. Panel a. examines how Turner paints the sun and sunlight in a more dynamic, realistic way than his predecessors. Turner's *Apullia in search of Appullus* (right, lower panel) is compositionally nearly-identical to Claude Lorrain's 1654 *Landscape with Jacob and Laban and Laban's Daughters* (right, upper panel) but includes a more subtle investigation of light and darkness. Panel b. shows a subset of Turner's cloud sketches possibly influenced by Luke Howard's cloud classification. Panel c. highlights four watercolors in Turner's 'Study of Sky' series following the 1815 Tambora volcanic eruption.
